# Solitary Fibrous Tumor of the Central Nervous System: A Report of Two Cases with Emphasis on Diagnostic Pitfalls

**DOI:** 10.1155/2024/3467025

**Published:** 2024-01-09

**Authors:** Absalom Mwazha, Nondabula Moyeni, Zuzile Zikalala, Gamalenkosi Bonginkosi Nhlonzi

**Affiliations:** ^1^Department of Anatomical Pathology, National Health Laboratory Service, Durban, South Africa; ^2^Discipline of Anatomical Pathology, University of KwaZulu-Natal, Durban, South Africa; ^3^Department of Neurosurgery, Inkosi Albert Luthuli Central Hospital, Durban, South Africa; ^4^Discipline of Neurosurgery, University of KwaZulu-Natal, Durban, South Africa; ^5^Department of Radiology, Dr. Pixley Ka Isaka Seme Memorial Hospital, Durban, South Africa; ^6^Discipline of Radiology, University of KwaZulu-Natal, Durban, South Africa; ^7^Department of Histopathology, Ampath Pathology Laboratories, Pietermaritzburg, South Africa

## Abstract

Solitary fibrous tumor (SFT) is a rare primary central nervous system neoplasm that usually presents as a dural-based mass. Awareness of the entity is limited by the rarity of the tumor which renders it prone to misdiagnosis. We present two cases of SFT located in the right parafalx and intraventricular region. The cases were classified as WHO grade 1 and grade 2, respectively. The present study discusses the radiological, histomorphological, and immunohistochemical features of SFT, with emphasis on potential diagnostic pitfalls that may lead to erroneous diagnosis.

## 1. Introduction

Solitary fibrous tumor (SFT) is a mesenchymal tumor of fibroblastic type showing a rich branching vascular pattern with a histopathological spectrum including tumors previously classified separately as meningeal solitary fibrous tumor and hemangiopericytoma (HPC) [1, 2]. The revised 4^th^ edition of the World Health Organization (WHO) classification of tumors of the central nervous system merged the two previously distinct diagnostic entities into a single category designated, solitary fibrous tumor/hemangiopericytoma [2]. The latest WHO classification has seen this category being designated as “solitary fibrous tumor” to align with the nomenclature used outside the central nervous system (CNS) [1]. This change is supported by a shared molecular hallmark, the chromosomal inversion at the 12q13 locus, which fuses the NGFI-A-binding protein 2 (*NAB2*) and the signal transducer and activator of transcription 6 (*STAT6*) genes [3]. *NAB2*-*STAT6* fusion variants have been identified and are grouped into two major variants: *NAB2* exon 4-*STAT6* exon 2/3 and *NAB2* exon 5/6/7-*STAT6* exon 16/17/18 based on clinicopathologic features [4–6]. SFTs with *NAB2 exon 4-STAT6 exon 2/3* fusion variants are often located in the pleuropulmonary area and are less cellular with low mitotic activity and abundant background collagen. *NAB2* exon 5/6/7-*STAT6* exon 16/17/18 fusion variants on the other hand are associated with SFT development in the meninges and deep soft tissue and are associated with hypercellularity and recurrent tumors [4–6]. Both fusion variants have been reported in the meningeal SFTs [1]. The *NAB2*:*STAT6* fusion results in diffuse and intense nuclear expression of STAT6 serving as a highly sensitive and specific immunohistochemical hallmark of SFT [1, 3].

The initial cases of SFT were reported in the pleura, and subsequently, extrapleural cases have been identified in various locations, including the lung, pericardium, mediastinum, soft tissue, and central nervous system [7]. Central nervous system SFT has been reported in isolated reports throughout different areas such as the tentorium cerebelli, frontal convexity, falx cerebri, cerebellopontine angle, suprasellar region, and ventricles [8, 9]. The majority of the reported cases in the central nervous system are dural-based, with a smaller portion presenting as subpial, intraparenchymal, or intraventricular tumors with no dural connection [10].

SFT needs to be distinguished from differentials, which are more common in this location which includes fibrous meningioma. The present study seeks to report on two cases of central nervous system SFT and discusses the radiopathological characteristics and potential diagnostic pitfalls.

## 2. Case Reports

### 2.1. Clinicoradiological Features

#### 2.1.1. Case 1

**Figure 1:**
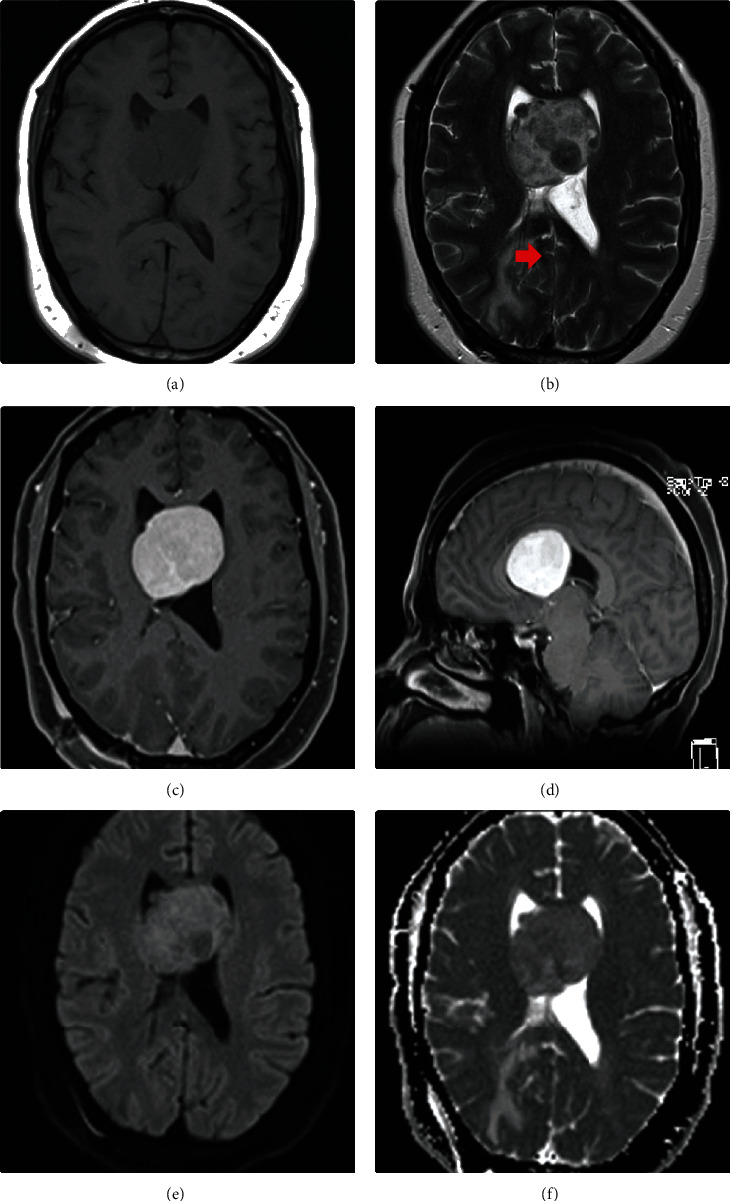
MRI images of a 57-year-old black female who presented with a history of headaches with no neurological deficits, after being involved in a pedestrian-vehicle accident. There is well well-circumscribed intraventricular lesion obstructing the foramen of Munro, with resultant hydrocephalus, thus VP shunt (red arrow) in situ. The lesion is isointense to the brain on T1 (a) and heterogeneous on T2 (b). Avid contrast enhancement is noted in (c) and (d). Diffusion-weighted imaging demonstrated restricted diffusion (e and f).

A 57-year-old black female presented at the base hospital with a history of a constant headache that started three months after a pedestrian-vehicle accident (PVA). Her headache was associated with dizziness and an unsteady gait but lacked vomiting or seizures. The patient was a known hypertensive on treatment. On clinical examination, she exhibited no neurological deficits. Precontrast brain computed tomography (CT) scans revealed a large midline third ventricular isodense mass with peripheral calcification that extended into both lateral ventricles obstructing the foramen of Monro leading to hydrocephalus. Preoperative magnetic resonance (MR) imaging showed a 4.6 × 4.5 × 4.1 cm well-demarcated intraventricular mass in the region of the foramen of Monro (Figure 1). The primary consideration was that of an intraventricular meningioma with subependymal giant cell astrocytoma, as an alternative. After admission, the patient underwent a right-sided paramedian craniotomy for gross tumor debulking surgery via an interhemispheric transcallosal approach. The pathological diagnosis was that of a solitary fibrous tumor (WHO grade I). Following recovery, she was transferred to the base hospital for further convalescence but unfortunately passed away two months postoperatively.

#### 2.1.2. Case 2

**Figure 2:**
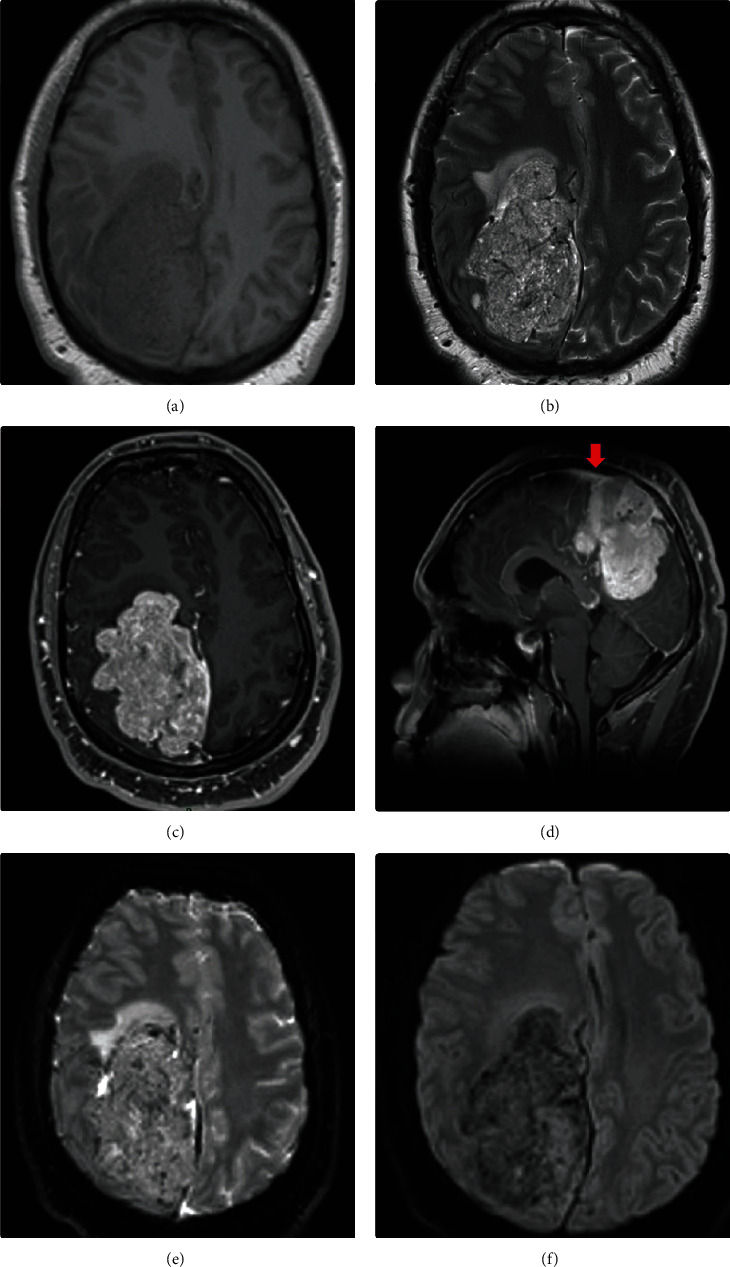
MRI images of a 22-year-old Asian male, presenting with blurred vision, headache, tinnitus, numbness of face, and left hemiplegia. (a) A large macrolobulated extra-axial mass arising from the dura in the supratentorial convexity. The mass has an intermediate signal similar to the brain on T1. (b) The mass demonstrates a heterogeneous “yin yang” appearance of low and high signal intensity on T2. (c) Diffuse contrast enhancement is seen and a (d) dural tail (red arrow) is present. (e, f) Areas of restricted diffusion are seen on diffusion-weighted imaging (DWI).

A 22-year-old Asian male, presented at the base hospital complaining of blurred vision, headache, tinnitus, numbness of the face, and left hemiplegia. He had no contributory medical history. MRI revealed a right-sided macrolobulated, extra-axial, posterior parafalcine mass measuring 8.4 × 6.5 × 5.7 cm (Figure 2). A preoperative diagnosis of parafalx meningioma was favored. Preoperative tumor embolization followed by tumor debulking which was performed in two stages due to intraoperative bleeding was done. The first stage involved right craniectomy and minimal tumor debulking (Simpson grade V). The histopathological analysis was reported as a WHO grade II atypical meningioma. The second stage was done one month later and involved debulking of the tumor (Simpson grade II) and insertion of a left frontal external ventricular drain. The histopathological analysis was reported as a WHO grade II solitary fibrous tumor. A review of the previous diagnosis also confirmed a WHO grade II solitary fibrous tumor. The patient was discharged to base hospital with right hemiplegia for rehabilitation. The patient received regular follow-up and rehabilitation. Unfortunately, the patient was lost to follow-up, and rehabilitation was interrupted during the COVID-19 (*coronavirus*) pandemic. He then presented at the base hospital, 18 months post the initial surgery, with a history of blurred vision, headache, and recurrent fits. MRI study showed a recurrent right parietal tumor with mixed solid and cystic areas. In addition, a large craniectomy defect in the biparietal regions and a left parietal region encephalocele was also observed. Tumor debulking (Simpson grade II) was subsequently done after the stabilization of the patient. His post-op recovery was plagued by recurrent wound breakdown and sepsis. The patient died 22 months after the initial presentation.

### 2.2. Histopathological Findings

**Figure 3:**
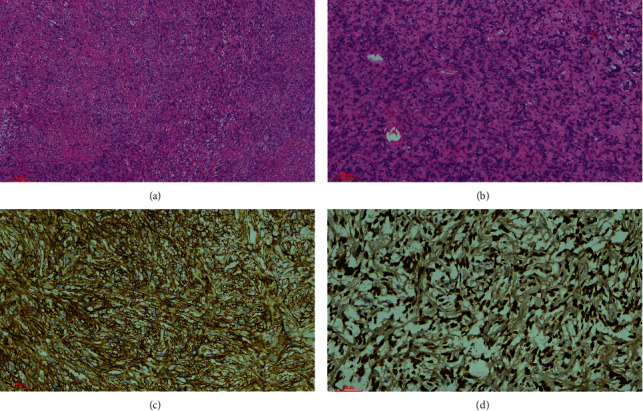
H&E solitary fibrous tumor WHO grade I. (a) Characteristic patternless growth pattern with oval to spindle-shaped tumor cells with a collagenous background (4x). (b) Tumor punctuated by thin-walled, branching vessels (10x). Immunohistochemically tumor cells show diffuse and strong staining with CD34 (c) and STAT6 (d) (40x).

In case 1, the tumor showed bland-appearing spindle cells, a collagenous background stroma, and prominent gaping thin-walled (staghorn) vessels. The spindle cells were arranged as short interlacing fascicles, sheets, and in areas showing storiform and patternless growth patterns (Figure 3). The neoplastic cells displayed indistinct cell borders, eosinophilic cytoplasm, round, oval, and elongated nuclei with finely dispersed chromatin and inconspicuous nucleoli. Nuclear pleomorphism, mitotic activity, or necrosis were not identified. Nuclear pseudoinclusions or psammomatous calcification was not evident.

**Figure 4:**
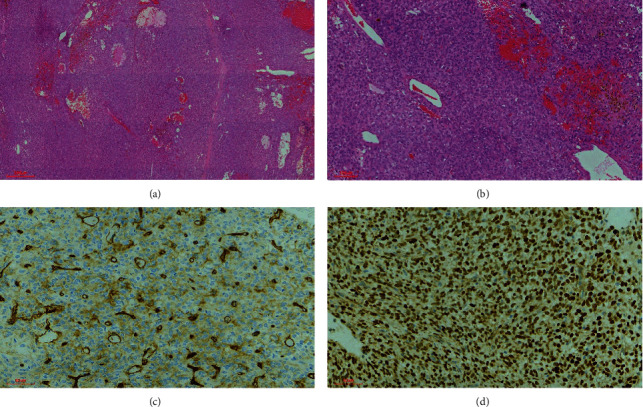
H&E solitary fibrous tumor WHO grade II. (a) Diffuse hypercellularity with minimal intervening stroma (4x). (b) Hypercellular tumor with staghorn-like vessels (10x). Immunohistochemically tumor cells show patchy and weak staining with CD34 (c) and show diffuse and strong staining with STAT6 (d) (40x).

Case 2 tumor showed similar histopathological features in all three biopsies. The tumor was hypercellular with a diffuse growth pattern punctuated by staghorn vessels and limited background stroma (Figure 4). The tumor showed pleomorphic, round, and oval nuclei and conspicuous nucleoli. In addition, hemorrhage and hemosiderin deposition were identified. Five mitotic figures were identified per ten high-power fields. Necrosis or calcifications were not evident.

### 2.3. Immunohistochemical Profile

All the cases showed immunoprofile typical of soft tissue counterparts. The neoplastic cells showed STAT6, CD34, BCL-2, and CD99 positivity (Figures 3 and 4). The low-grade SFT (WHO grade 1) case showed *diffuse* and strong CD34 *staining* compared to the high-grade (WHO grade 2) case, which showed a patchy and weak *staining* pattern with CD34.

There was no immunoreactivity with epithelial membrane antigen (EMA), S-100 protein, cytokeratins (CK), progesterone receptor, synaptophysin, glial fibrillary acidic protein (GFAP), desmin, and smooth muscle actin (SMA) in both cases. Case two was also negative for HMB-45 and Melan A. The Ki-67 proliferation index was less than 2% in case 1 and approximately 10% in case 2.

## 3. Discussion

The identification of histopathological and immunohistochemical features of SFT is crucial for appropriate diagnosis of this entity. Central nervous system SFT is a rare neoplasm accounting for <1% of all primary CNS tumors and has undergone recent reclassification [1, 2]. The current terminology for SFT is now similar to that used outside the CNS (e.g., in the soft tissue, pleura, and other visceral sites). The use of the term solitary fibrous tumor/hemangiopericytoma or hemangiopericytoma in the CNS is no longer recommended. Solitary fibrous tumors can be grouped into 3 grades based on mitotic activity and the presence or absence of necrosis [1, 11, 12]. The present case series presents two cases of SFT, one of which was initially misdiagnosed as an atypical meningioma.

SFT imaging features can resemble other more common CNS tumors such as meningiomas, schwannomas, dural metastasis, and primary dural lymphoma. At present, no specific features on CT or MRI can be used to distinguish SFT from meningiomas [13]. Pretreatment differentiation is essential as the behavior and treatment of these tumors are different. The two cases in the current studies had meningioma as the favored radiological diagnosis before they were proved otherwise on histopathology.

Magnetic resonance imaging (MRI) is the imaging modality of choice. The CNS solitary fibrous tumors have an intermediate signal similar to the brain on T1. They are iso-to-hypo intense to the brain on T2 and may typically demonstrate a heterogeneous “yin yang” appearance of low and high signal intensity. Typically, avid contrast enhancement is seen. A dural tail may be present. Areas of restricted diffusion are commonly seen on diffusion-weighted imaging (DWI) [14–16]. Magnetic resonance spectroscopy (MRS) demonstrates myo-isotonol, lipid, and lactate elevation [16, 17].

The solitary fibrous tumors may be difficult to differentiate from meningioma on imaging as they share common features such as the presence of a dural tail [1, 13]. Solitary fibrous tumors, however, rarely demonstrate calcifications and hyperostosis of adjacent bone [18]. Case 1 showed calcifications which also swayed preoperation diagnosis towards a meningioma. Myo-isotonol elevation on advanced MRI such as MR spectroscopy may be useful [16, 17]. Research exploiting diffusion-weighted imaging susceptibility-weighted imaging, and deep learning artificial intelligence (AI) is ongoing [19].

On histopathology, SFT shows a range of phenotypes ranging from hypocellular to hypercellular phenotypes. The more classic hypocellular phenotype displays short spindle and oval-round cells arranged in a “patternless pattern” but occasionally arranged in fascicles, with alternating thick bands of hyalinized collagen and thin-walled branching hemangiopericytoma-like (staghorn) vessels [8]. The nuclei are round or oval, with moderately dense chromatin and inconspicuous nucleoli. Mitotic activity is generally not seen. These are considered benign and classified as WHO grade 1 [1, 9, 11]. Pseudoinclusions characteristic of meningiomas are not observed [1, 8]. It is, however, important to note that local recurrences, malignant progression, and metastasis have been reported in cases that would otherwise be categorized as benign (WHO grade 1) [10, 20].

The hypercellular phenotype is characterized by hypercellularity, with oval-round cells arranged in a haphazard pattern with minimal intervening stroma, and is considered malignant. These are generally treated by surgery and adjuvant radiotherapy [21]. Mitotic activity and necrosis are common, whilst calcifications are not seen [22]. Tumors with a mitotic count ≥ 5 mitoses/10 HPF without necrosis are classified as grade 2 whilst those with ≥5 mitoses/10 HPF with necrosis are classified as grade 3 [1, 9, 11].

SFT is typically diffusely positive for CD34, CD99, and STAT6. STAT6 immunohistochemistry has a very high specificity and sensitivity for detecting *NAB2-STAT6* fusion and is considered a definitive marker of this entity [1, 3]. BCL2, EMA, SMA, and progesterone receptor positivity may rarely be identified as a focal finding [22–24]. The median Ki-67 proliferation index median is 5% in cases showing classic hypocellular phenotype and 10% in cases showing hypercellular phenotype [1, 2].

Fibrous meningioma and myofibroblastoma need to be differentiated from hypocellular SFT [8, 25] whilst anaplastic and atypical meningioma may show histopathological features similar to hypercellular SFT [24]. Fibrous meningioma characteristically expresses EMA and is negative for CD34 and nuclear STAT6 expression. Meningeal myofibroblastoma typically expresses CD34, desmin, and SMA and is negative for EMA, S100, and nuclear STAT6 expression [25]. Anaplastic and atypical meningioma may show loss of EMA staining but are negative for CD34 and STAT6 [24]. It is important however to note that meningiomas may show weak nuclear and/or cytoplasmic positivity for STAT6 but not strong isolated nuclear immunostaining [2]. Dural-based Ewing sarcoma/primitive neuroectodermal tumor shares the hypercellularity and CD99 positivity of hypercellular SFT phenotype but lacks nuclear STAT6 staining and is characterized by *EWSR1* gene rearrangement [26]. Monophasic synovial sarcomas can resemble hypercellular SFT due to CD99 and BCL2 positivity. Positive staining with cytokeratin, EMA, and TLE1 and lack of nuclear STAT6 and/or FISH analysis for the presence of *SYT* gene rearrangement support this diagnosis [27]. Rarely, malignant peripheral nerve sheath tumor (MPNST) occurs in the meninges and may resemble the hypercellular SFT phenotype [28]. However, MPNST is usually negative for CD34 and STAT6 and may show focal expression of S100 protein and SOX10.

## 4. Conclusion

In summary, we report here two rare cases of SFT in the central nervous system. The awareness of the existence of this tumor type, recent reclassification, and the differential diagnosis have relevance for neurosurgeons, radiologists, and pathologists.

## Data Availability

The data supporting the conclusions of this study are found within the article and by consulting the works cited.
